# The microstructural change of the brain and its clinical severity association in pediatric Tourette syndrome patients

**DOI:** 10.1186/s11689-023-09501-0

**Published:** 2023-10-25

**Authors:** Chia-Jui Hsu, Lee Chin Wong, Hsin-Pei Wang, Yi-Chun Chung, Te-Wei Kao, Chen-Hsiang Weng, Wen-Chau Wu, Shinn-Forng Peng, Wen-Yih Isaac Tseng, Wang-Tso Lee

**Affiliations:** 1 Department of Pediatrics, National Taiwan University Hsin-Chu Hospital Hsin-Chu Branch, Hsin-Chu, Taiwan; 2 Graduate Institute of Brain and Mind Sciences, National Taiwan University College of Medicine, Taipei, Taiwan; 3 Department of Pediatrics, Cathay General Hospital, Taipei, Taiwan; 4 Department of Pediatrics, National Taiwan University Children’s Hospital, Taipei, Taiwan; 5 Graduate Institute of Clinical Medicine, National Taiwan University College of Medicine, Taipei, Taiwan; 6 Department of Pediatrics, National Taiwan University Hospital Yunlin Branch, Yunlin, Taiwan; 7 Institute of Medical Device and Imaging, National Taiwan University College of Medicine, Taipei, Taiwan; 8 Department of Radiology, National Taiwan University Hospital and College of Medicine, National Taiwan University, Taipei, Taiwan; 9 Department of Pediatrics, National Taiwan University Hospital and College of Medicine, National Taiwan University, Taipei, Taiwan

**Keywords:** Diffusion spectrum imaging, False discovery rate, Frontostriatal, Pediatric neurology, Gilles de la Tourette syndrome

## Abstract

**Background:**

Gilles de la Tourette syndrome (GTS) is a prevalent pediatric neurological disorder. Most studies point to abnormalities in the cortico-striato-thalamocortical (CSTC) circuits. Neuroimaging studies have shown GTS’s extensive impact on the entire brain. However, due to participant variability and potential drug and comorbidity impact, the results are inconsistent. To mitigate the potential impact of participant heterogeneity, we excluded individuals with comorbidities or those currently undergoing medication treatments. Based on the hypothesis of abnormality within the CSTC circuit, we investigated microstructural changes in white matter using diffusion spectrum imaging (DSI). This study offers the first examination of microstructural changes in treatment-naïve pediatric patients with pure GTS using diffusion spectrum imaging.

**Methods:**

This single-center prospective study involved 30 patients and 30 age- and gender-matched healthy volunteers who underwent sagittal T1-weighted MRI and DSI. We analyzed generalized fractional anisotropy, mean diffusivity, axial diffusivity, and radial diffusivity.

**Results:**

No significant differences were observed in mean diffusivity and axial diffusivity values between the two groups. However, the patient group exhibited significantly higher generalized fractional anisotropy values in the right frontostriatal tract of the dorsolateral prefrontal cortex, the right frontostriatal tract of the precentral gyrus, and bilateral thalamic radiation of the dorsolateral prefrontal cortex. Additionally, the generalized fractional anisotropy value of the right frontostriatal tract of the precentral gyrus is inversely correlated with the total tic severity scores at the most severe condition.

**Conclusion:**

Treatment-naïve pediatric GTS patients demonstrated increased connectivity within the CSTC circuit as per diffusion spectrum imaging, indicating possible CSTC circuit dysregulation. This finding could also suggest a compensatory change. It thus underscores the necessity of further investigation into the fundamental pathological changes in GTS. Nevertheless, the observed altered connectivity in GTS patients might serve as a potential target for therapeutic intervention.

**Supplementary Information:**

The online version contains supplementary material available at 10.1186/s11689-023-09501-0.

## Introduction

Tic disorders, characterized by rapid and stereotyped movements or sounds, are prevalent pediatric neurological conditions. Gilles de la Tourette syndrome (GTS) is a particular form of tic disorder that typically emerges during childhood, affecting approximately 0.1–6% of the human population [1–3]. GTS is chiefly defined by intermittent motor and vocal tics that fluctuate in severity. Beyond these core symptoms, around 90% of GTS patients experience additional conditions such as attention-deficit hyperactivity disorder, obsessive–compulsive disorder (OCD), depression, or sleep disorders [4–6].

The pathogenesis of GTS remains unclear. However, most research emphasizes basal ganglion disinhibition and related anomalies in the cortico-striatal-thalamocortical circuit [3, 7–10]. Diffusion tensor imaging (DTI) is a specialized MRI technique used to study microstructural changes in the brain’s white matter in vivo [11, 12]. Prior DTI research has identified altered connectivity within CSTC circuits, encompassing the basal ganglia, motor cortex, supplementary motor cortex, sensorimotor cortex, and corticospinal tracts [8, 13–15].

Nevertheless, microstructural alterations in GTS patients may differ between adults and children. Adult patients often exhibit heightened connectivity in the post- and precentral gyrus, left supplementary motor area, cingulate cortex, inferior parietal cortex, and frontal cortex [8, 13]. Alterations have also been observed in cortico-striatal and thalamocortical pathways and deep gray matter structures, including the putamen, amygdala, and nucleus accumbens [8, 15]. Conversely, connectivity is typically lower in pediatric GTS patients [16]. DTI studies with this younger cohort have reported diminished connectivity between the caudate nucleus and the left anterior-dorsolateral-frontal cortex [16]. Decreased fractional anisotropy values and increased radial diffusivity (RD) values have also been recorded in the corpus callosum, somatosensory cortex, primary motor cortex, and anterior thalamic radiation [17, 18]. Despite these age-dependent discrepancies, the collaborative research supports the idea of abnormal CSTC circuit connectivity in GTS patients.

Previous studies indicate microstructural and hemodynamic changes across multiple intracranial regions in GTS patients. Yet, the fundamental cerebral pathology’s core location remains undefined. The primary aim of this study is to identify the significantly altered intracranial microstructure implicated in GTS pathogenesis. As up to 90% of GTS patients exhibit comorbidities, it is essential to account for this in the research. Many earlier studies did not exclude patients with comorbidities, which introduces confounding variables that must be addressed when evaluating GTS patients’ microstructural changes and identifying the primary aberrant brain region associated with this condition. To reduce the effects of medication or comorbidities, our research focuses on recruiting unmedicated pediatric individuals with pure GTS, thus confronting the high heterogeneity issue prevalent in previous studies’ enrollment criteria. Through this approach, we aim to deepen our understanding of GTS pathogenesis and uncover novel pathways to further elucidate its underlying mechanisms.

## Methods and materials

### Participants

Given that GTS is primarily a neurodevelopmental disorder with onset in childhood, it is noteworthy that fewer than half of the patients continue to exhibit symptoms into adulthood. For this reason, we believe that pediatric GTS patients may offer a more representative sample. We enrolled patients from the northern part of Taiwan, including the National Taiwan University Children’s Hospital, Taipei, Taiwan. The inclusion criteria of patient groups are treatment-naïve pediatric GTS patients (ranging 6–16 years) without other psychiatric comorbidities, developmental delay, or severe underlying medical diseases. The diagnosis of GTS and other psychiatric comorbidities was based on the diagnostic criteria from the *Diagnostic and Statistical Manual of Mental Disorders, 5th Edition* [19]. The typically developing children without history of tic disorder and neurological, psychological, or underlying medical diseases were enrolled as healthy control (HC). For all participants, they were interviewed in detail about their birth history, developmental milestones, underlying medical disease, or other neurological disease. We also used questionnaires to objectively diagnose psychological comorbidities. The questionnaires we used included the Chinese version of Obsessive–Compulsive Inventory-Revised [20], the Chinese version of Children’s Depression Inventory [21], the Chinese version of migraine disability assessment score [22], the Chinese version of Edinburgh Handedness Inventory [23], and the Chinese version of the Swanson, Nolan, and Pelham, version IV [24]. After interviewing, all of them would receive intelligence test performed by pediatric psychologist with the Chinese version of Wechsler Intelligence Scale for Children-IV [25]. The disease severity of GTS was measured by pediatric neurologists with (YGTSS) [26].

### Image data acquisition and analysis

Participants and their parents or guardians provided informed consent and signed a checklist of MRI contraindications. All scans were acquired using a Siemens Tim Trio 3 T with 32-channel head coils. High-resolution T1-weighted imaging was performed using a 3D magnetization-prepared rapid gradient echo sequence (*TR* = 2000 ms, *TE* = 3 ms, flip angle = 9°, resolution = 1 × 1 × 1 mm^3^, total number of slices per slab = 208). The diffusion spectrum imaging (DSI) utilized a pulsed-gradient spin-echo diffusion echo-planar imaging sequence with twice-refocused balanced echo (*TR* = 9600 ms, TE = 130 ms, in-plane spatial resolution = 2.5 × 2.5 mm^2^, slice thickness = 2.5 mm, *FOV* = 200 × 200 mm^2^, matrix size = 80 × 80 × 56 slices, bmax = 4000 s/mm^2^).

The analysis method for the whole brain neural fiber tracts was termed tract-based automatic analysis (TBAA) [11]. TBAA is a template containing 76 neural fiber tracts reconstructed by high-quality diffusion spectrum image and T1-weighted image. It is reconstructed on the NTU-DSI-122 template which is a standard coordinate system with high-quality resolution and constructed 76 neural fiber bundles [27]. We selected four DSI parameters for data analysis. The interpretation of generalized fractional anisotropy (GFA) is similar to the interpretation of fractional anisotropy in general DTI studies, in that it indicates the degree of water diffusion anisotropy without orientation information. Mean diffusivity (MD), radial diffusivity (RD), and axial diffusivity (AD) value provide the information of diffusion direction of each voxel in three vectors [28]. The TBAA procedure registered the DSI data with T1-weighted imaging to create a study-specific template and then registered it to the NTU-DSI-122 template separately. This two-step registration strategy combined the anatomical information from high-resolution T1-weighted images with microstructural diffusion indices provided by DSI.

### Statistical analysis

Demographic characteristics were presented numerically and as a percentage for both patient and HC groups, with the *p*-value calculated from an independent *t*-test. The scores of YGTSS were subdivided into six subscores: motor tics scores, vocal tics scores, total tics scores, motor tics scores at the present stage, and the most severe condition.

Given that intracranial regions are widely influenced in GTS patients according to previous studies, we compared all 76 neural fiber tracts between the patient group and the HC group. According to literature reviewing, abnormality of cerebral regions of CSTC circuit is the main finding in GTS patients so we chose eight frontal-striatal tracts and ten thalamic radiations involved in CSTC circuit for further analysis. We selected mean GFA, MD, AD, and RD as parameters for microstructural changes generated from TBAA and analyzed them using an independent *t*-test. Corrections were made for multiple comparisons across selected neural fiber tracts in the *q*-value using the Benjamini–Hochberg false discovery rate (FDR).

We selected the neural fiber tracts showing more significant differences between patient and HC groups for further evaluation. We used simple and multiple linear regression to test the correlation between DSI results, demographic characters, and disease severity. All statistical analyses were computed using IBM SPSS v22 (SPSS Inc., Chicago, IL, USA). A *p*-value less than 0.05 was considered statistically significant.

## Results

### Demographic data

**Fig. 1:**
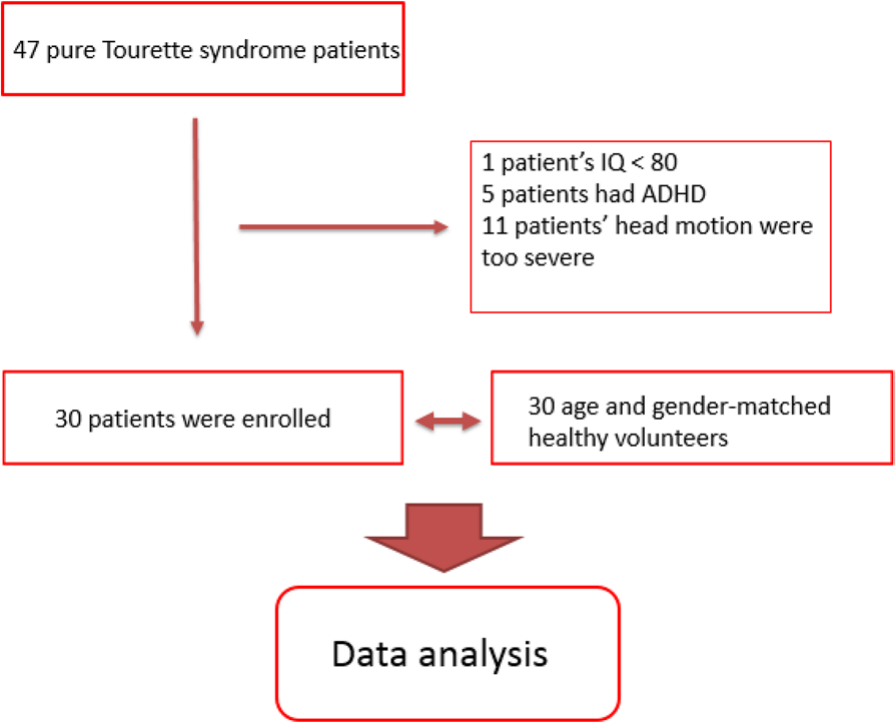
The algorism of participant enrollment

A total of 47 GTS patients were initially enrolled in our study. However, due to comorbidities or lack of cooperation, 17 patients were excluded. Thus, only 30 treatment-naïve GTS patients were selected for data analysis. We also utilized DSI data from 30 age- and gender-matched healthy volunteers as the control group. The participant enrollment algorithm is illustrated in Fig. 1. The dropout number indicates missing images during DSI scanning and is indicative of the degree of motion artifacts during MRI scanning. These confounding factors showed no statistical difference between the two groups (Table 1). The tic severity scores were assessed according to the YGTSS. Our study only recorded the total tic severity scores, with a maximum of 50 points, as the functional impairment rating can easily be influenced by individual psychosocial status. The clinical tic severity scores of the patient group are listed in Table 1.

**Table 1:** Demographic data of TS group and HC group and the clinical severity of patients group

	**TS group**	**HC group**	***p*-value**
Number	30	30	
Gender (M/F)	22/8	22/8	1.0
Age (yrs) (mean ± SD)	9.28 ± 1.94	9.41 ± 1.91	0.657
FSIQ (mean ± SD)	106.9 ± 9.31	109.6 ± 9.21	0.355
Dropout number (mean ± SD)	16.4 ± 15.5	23.2 ± 28.1	0.255
Disease duration (yrs) (mean ± SD)	2.1 ± 1.4	NA	
VTS at present (mean ± SD, max: 25)	5.1 ± 4.9	NA	
VTS at the most severe condition (mean ± SD, max: 25)	6.5 ± 4.3	NA	
MTS at present (mean ± SD, max: 25)	10.3 ± 3.9	NA	
MTS at the most severe condition (mean ± SD, max: 25)	11.6 ± 3.6	NA	
TTS at present (mean ± SD, max: 50)	15.3 ± 6.3	NA	
TTS at the most severe condition (mean ± SD, max: 50)	18.0 ± 5.7	NA	

*HC* healthy control, *Max* maximum scores, *MTS* motor tics score, *SD* standard deviation, *TS* Tourette syndrome, *TTS* total tics scores, *VTS* vocal tics scores

### Whole-brain comparison

In the whole-brain comparison, the primary differences between patients and healthy controls were in the GFA and RD values. The GFA value in DSI represents the change in microstructural integrity. Of the total 76 neural fiber tracts, 16 showed significant *p*-value in GFA, including the right frontal-striatal tract of the dorsolateral prefrontal cortex and the left frontal-striatal tract of the ventrolateral prefrontal cortex. No neural fibers presented a significant *q*-value. However, it was observed that GFA values were higher in the patient group than in the HC group across all affected neural fiber tracts, suggesting increased connectivity in the patient group. Moreover, most affected neural fiber tracts correlated with the thalamus and prefrontal cortex. This result aligns with the hypothesis of abnormality within the CSTC circuit in GTS pathogenesis.

RD values typically represent the degree of myelination. In our results, ten neural fiber tracts had a significant *p*-value for RD, including the right frontal-striatal tract of the DLPFC and the corpus callosum of the DLPFC. No neural fibers showed a significant *q*-value after multiple comparison. The RD values of affected neural fiber tracts were higher in the patient group than in the HC group, suggesting hypermyelination of affected neural fiber tracts in GTS patients. The distribution of affected neural fiber tracts in RD highly overlapped with GFA value-affected neural fiber tracts. It indicated higher connectivity in GTS patients within the CSTC circuit (Supplementary Table 1).

### DSI analysis within CSTC circuit

**Fig. 2:**
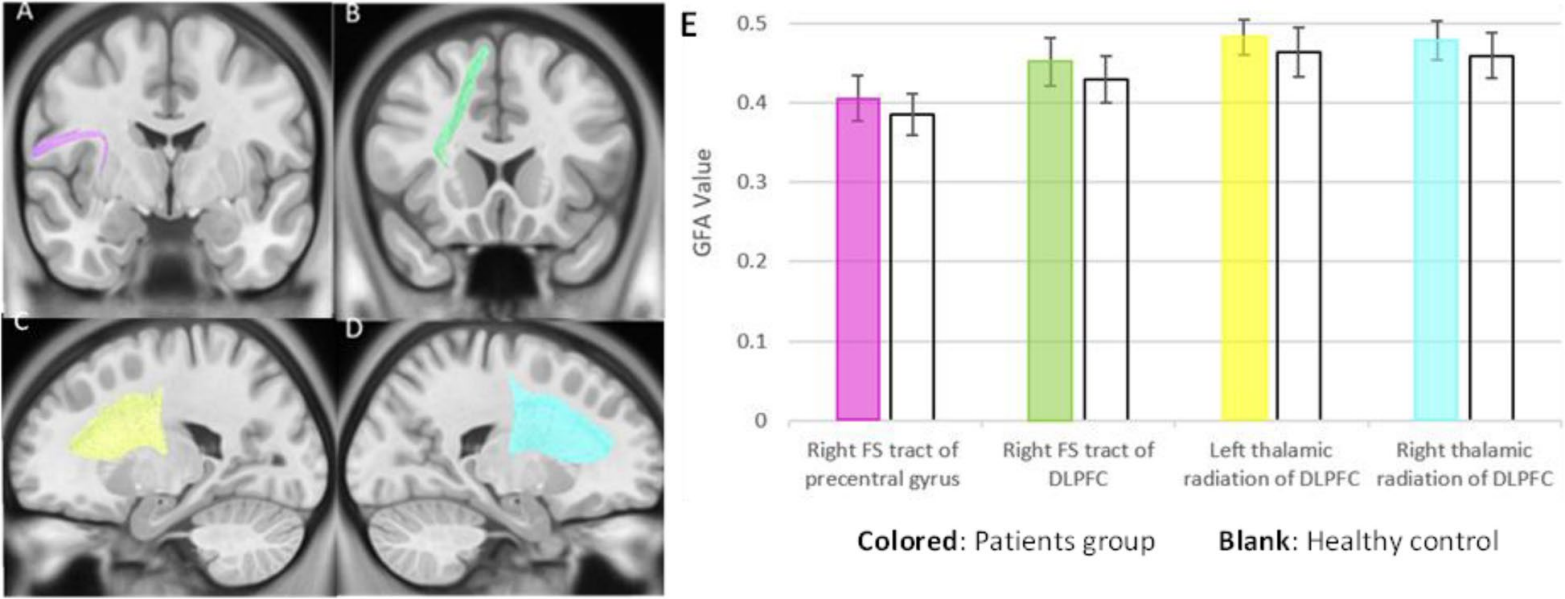
The main affected neural fiber tracts in GTS patients. **A** Right frontal-striatal tract of precentral gyrus. **B** Right frontal-striatal tract of dorsolateral prefrontal cortex. **C** Left thalamic radiation of dorsolateral prefrontal cortex. **D** Right thalamic radiation of dorsolateral prefrontal cortex. **E** Comparison: patient group versus healthy group in four neural fiber tracts

Existing literature consistently points to dysfunction in the CSTC circuit as a primary concern in GTS. Abnormalities in neurotransmitter levels and microstructural and/or hemodynamic changes within this circuit have been reported in numerous studies [29–31]. Under the hypothesis of abnormal connectivity of the CSTC circuit in GTS patients, we further analyzed changes in the neural fiber tracts within this circuit. Eighteen neural fiber tracts were selected as components of the CSTC circuits, including eight frontostriatal (FS) tracts and ten thalamic radiations. The most noticeable differences were observed in GFA and RD values. Eight neural fiber tracts showed a significant *p*-value for an increase in GFA value; four remained significant after multiple comparison corrections. These four neural fiber tracts were the right FS tract of DLPFC, the right FS tract of the precentral gyrus, and bilateral thalamic radiation of DLPFC (Fig. 2). In the RD analysis, none of the neural fiber tracts survived correction for multiple comparisons. However, a consistent trend indicated a decrease in RD values within the patient group, although these differences did not reach statistical significance after correction for multiple comparisons. These findings suggest a potential association between the patient group and reduced RD values, warranting further investigation to confirm this conclusively. Overall, these findings suggest increased functional connectivity within the CSTC circuit in individuals diagnosed with GTS, indicating heightened interregional communication and synchronized activity among key nodes within the CSTC network (Supplementary Table 2).

### The correlation between clinical tics severity and DSI results

**Fig. 3:**
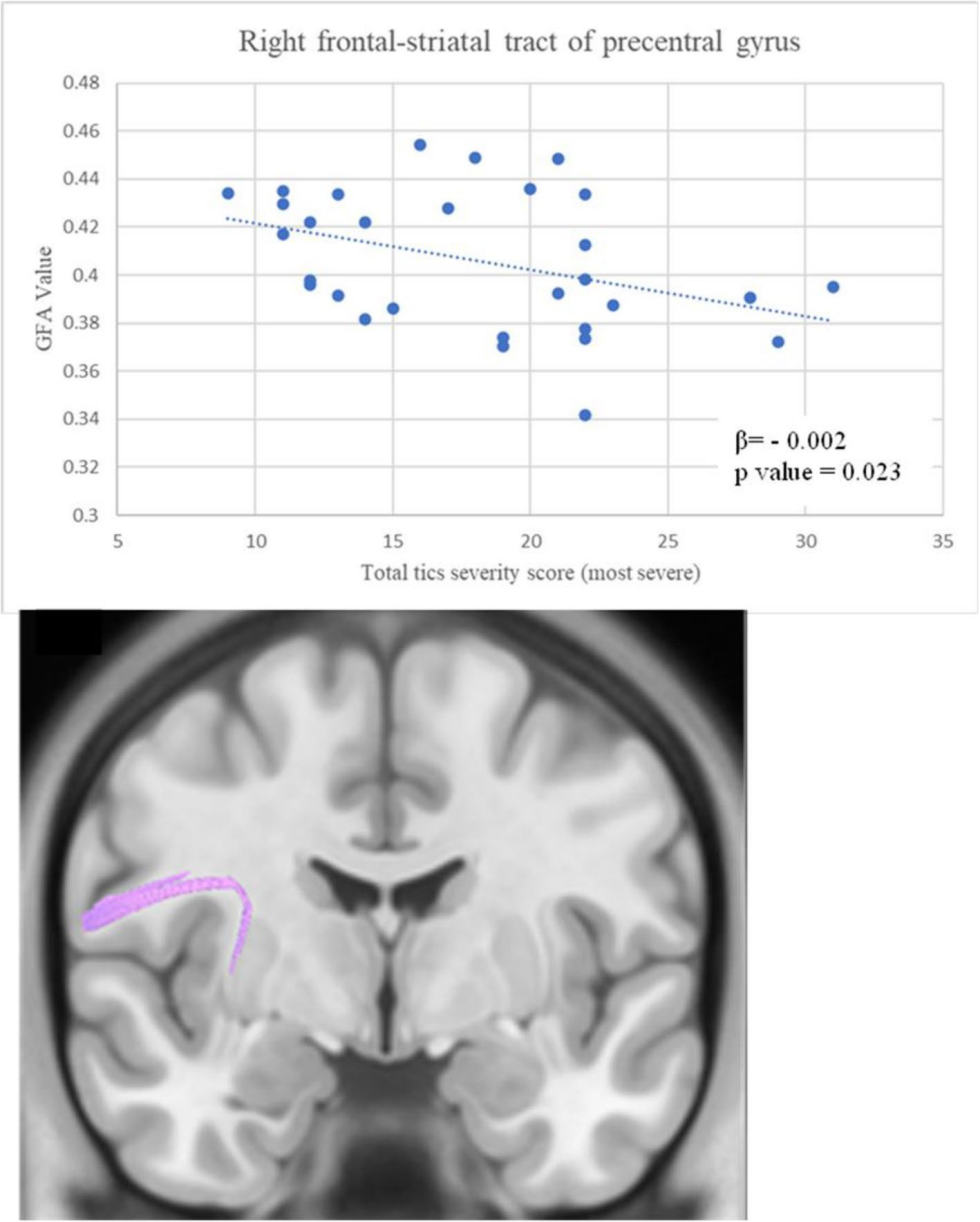
The correlation between clinical tics severity and GFA value in right frontal-striatal tract of precentral gyrus

The tic severity scores consisted of motor tics scores, vocal tics scores, and total tics scores. In our study, we recorded the tic severity scores when performing the MRI and the scores when their symptoms were most severe. We initially found significant differences in increased GFA values over FS tracts and thalamic radiations, with a trend of decreasing RD values. Therefore, we sought a correlation between tic severity scores and GFA and RD values of FS tracts and thalamic radiations. The normality test of GFA and RD value of selected neural fiber tracts showed normal distribution. After performing simple and multiple linear regression analyses, the GFA value of the right frontal-striatal tract of the precentral gyrus showed a significant negative correlation with total tic scores at their most severe condition (*β*: − 0.002, *p* = 0.023) (Table 2 & Fig. 3). The RD value of the left thalamic radiation of DLPFC displayed a trend of positive correlation with current motor tic scores (*β*: 0.001, *p* = 0.0576) (Table 2).

**Table 2:** The association between DSI and the potential risk factors

	Model 1			Model 2		
	β	(95% *CI*)	*p*-value	β	(95% *CI*)	*p*-value
GFA R’t FS tract of precentral gyrus						
Age	0.005	(0.001 ~ 0.009)	0.011	0.004	(− 0.001 ~ 0.009)	0.117
Gender	0.004	(− 0.013 ~ 0.021)	0.601			
Disease duration	0.003	(− 0.005 ~ 0.011)	0.444			
Motor tics score	− 0.001	(− 0.003 ~ 0.001)	0.328			
Vocal tics score	− 0.001	(− 0.004 ~ 0.002)	0.373			
Total tic score	− 0.001	(− 0.003 ~ 0.001)	0.168			
Motor tic score (most severe)	− 0.002	(− 0.004 ~ 0.000)	0.106			
Vocal tics score (most severe)	− 0.002	(− 0.005 ~ 0.001)	0.172			
Total tics score (most severe)	− 0.002	(− 0.004 ~ 0.000)	0.033	− 0.002	(− 0.004 ~ − 0.0002)	0.023*
RD L’t thalamic radiation of DLPFC						
Age	− 0.005	(− 0.008 ~ − 0.002)	0.002	− 0.003	(− 0.007 ~ 0.000)	0.0763
Gender	− 0.006	(− 0.020 ~ 0.009)	0.445			
Disease duration	− 0.003	(− 0.008 ~ 0.003)	0.280			
Motor tics score	0.001	(0.000 ~ 0.003)	0.043	0.001	(0.000 ~ 0.003)	0.0576
Vocal tics score	− 0.001	(− 0.003 ~ 0.001)	0.369			
Total tic score	0.001	(0.000 ~ 0.002)	0.260			
Motor tic score (most severe)	0.002	(0.000 ~ 0.003)	0.064			
Vocal tics score (most severe)	− 0.001	(− 0.003 ~ 0.001)	0.568			
Total tics score (most severe)	0.001	(− 0.001 ~ 0.002)	0.308			

Model 1, estimated by simple linear regression. Model 2, estimated by multiple linear regression. *t-value =  − 2.407, adjusted R square = 0.169. *DLPFC* dorsolateral prefrontal cortex, *FS* frontal-striatal, *L’t* left, *R’t* right

## Discussion

In the present study, patients with GTS had higher connectivity over CSTC circuits with increased GFA values. In several studies on pediatric patients, the connectivity has been reported to be generally lower in patients with GTS [16, 18, 32, 33]; however, our study showed increased connectivity in the patient group. Moreover, the participants included in previous studies varied, and only one of those studies included patients with GTS who had received no treatment [34]. However, this study only analyzed the abnormality of corpus callosum and did not discuss about other brain regions [34]. ADHD is the most common comorbidity in patients with GTS, and most studies on pediatric GTS enroll patients with GTS having ADHD. Decreased FA value over cingulate gyrus, corticospinal tracts, and frontal lobe and increased RD value over corpus callosum and thalamic radiation have been reported in pediatric patients with ADHD [35, 36]. For patients with GTS, the results of microstructural change might be influenced by the comorbidities. In the present study, most affected neural fiber tracts were related to frontal cortex, especially over DLPFC and VLPFC. The involvement of frontal cortex has been widely mentioned in previous studies [7, 8, 30, 31]. Functional MRI studies have revealed increased activities over prefrontal cortex during motor execution and motor imagination [37–39]. Brain volumetric studies have revealed that both gray and white matter volumes over prefrontal cortex decrease in patients with GTS [30, 33, 40]. The NIRS study revealed that patients with GTS had altered hemodynamic response over prefrontal cortex during Stroop color-word task [41]. Based on these findings, the involvement of frontal cortex in patients with GTS in the present study is not surprising. DLPFC is involved in working memory and decision-making processing [42, 43]. VLPFC is involved in working memory, post-decision processing, and motor inhibition during sudden conditions [44–46]. In patients with GTS, impairment of working memory function, including spatial recognition, story recall, and procedural memory, has been reported [47, 48]. Therefore, working memory impairment is one of the characteristics of GTS. This phenomenon also reflected the involvement of DLPFC and VLPFC in patients with GTS.

The results of the present study also suggested the involvement of basal ganglion and thalamus, which are core components of CSTC circuits. Altered activity of putamen was observed in fMRI before tics onset [49]. In a previous study, resting-state fMRI showed abnormal spontaneous neuronal activity of cingulate gyrus, putamen, and thalamus [50]. DTI studies have revealed increased AD value over putamen and thalamic radiation and increased RD value over right thalamus in pediatric patients [33, 51]. In the present study, the affected neural fiber tracts were all connected with striatum or thalamus. It is reasonable that the affected bundles were components of CSTC circuits. Increased connectivity of frontostriatal tracts might influence both direct and indirect pathways in CSTC circuits. The net effect of the direct pathway is to increase motor activity, whereas that of the indirect pathway is to inhibit motor function. In patients with GTS, elevated D2-binding ability has been observed on PET [52, 53], indicating that the effect of the indirect pathway is more dominant than that of the direct pathway. Increased connectivity of frontostriatal tracts might cause more motor inhibition via the indirect pathway. This phenomenon may suggest the compensatory mechanism in patients with GTS.

Clinical observations show that individuals with GTS have a negative correlation between their most severe tic scores and the GFA value over the precentral gyrus’s right FS tract. This contradicts a study by Liu et al. who reported a positive correlation between tic severity scores and the AD value over the right anterior thalamic radiation and right cingulum bundles [33]. However, our study did not find a correlation between tic severity scores and AD value.

Several DTI studies have shown a negative correlation between the FA value and tic severity scores, as well as a positive correlation between the RD value and tic severity scores in various white matter regions such as the thalamus, anterior thalamic radiation, superior longitudinal fasciculus, and forceps major [13, 18, 32]. Our study also found similar correlations between microstructural change and clinical severity association. However, our results show an increased GFA value in individuals with GTS, with a negative correlation between GFA value and total tic severity scores within CSTC circuits. One hypothesis to explain this phenomenon is that individuals with GTS may have a compensatory increase in connectivity, even though they exhibit fewer clinical symptoms. Our results suggest that this compensatory mechanism may begin as early as childhood.

Interestingly, our study also reveals that the GFA value may better correlate with total tic severity in the past rather than the present. This indicates that some microstructural changes may persist even when an individual’s clinical severity has altered.

### Limitation

Of course, our results should be considered alongside a few limitations. First, recollecting symptoms at their most severe condition could introduce recall bias. Instead of scoring the severity of tics based on current observations, tic severity scores were largely rated according to parents’ reports if the symptoms of the individuals with GTS were not at their most severe condition at the time of the interview. To mitigate recall bias, pediatric neurologists thoroughly explained tic symptoms and conducted careful interviews with parents and patients to reconstruct the symptoms at their most severe condition.

Second, the time when the most severe condition occurred varied among individuals. To date, no studies have investigated the longevity of microstructural changes when the intracranial microstructure is affected by a disease or how connectivity might change as disease severity fluctuates. It also remains uncertain whether current MRI scanning data accurately reflect the condition of the most severe phase in every participant.

Third, our study’s sample size may not offer enough statistical power to examine all 76 neural fiber tracts, but our primary focus is on the 18 neural fibers within the CSTC circuit. Given that treatment-naïve, pure GTS patients are relatively rare, future research with a larger sample size is necessary for a more precise understanding of intracranial microstructural changes in GTS patients.

## Conclusion

Our research is the first to analyze the brain structure of children with GTS who have not yet received treatment, using DSI. We found that the GFA value in the right FS tract of DLPFC, right FS tract of the precentral gyrus, and bilateral thalamic radiation of DLPFC was significantly increased. This indicates that CSTC circuits are involved in people with GTS, and that there is higher connectivity in children with GTS. We also found that the GFA value in the right FS tract of the precentral gyrus is negatively correlated with the severity of tic symptoms. This suggests that people with less severe symptoms have better compensation within the CSTC circuit and thus exhibit higher connectivity than those with more severe symptoms. Our results also indicate that the GFA value of the right FS tract of the precentral gyrus may reflect the most severe condition, implying that changes in brain structure may persist for a certain period even after clinical severity has changed. These findings provide potential targets for intervention in future research.

### Supplementary Information

**Additional file 1: Supplementary Table 1.** The result of whole brain comparison. **Supplementary Table 2.** The result of comparison within CSTC circuit. The listed neural fiber tracts include only those with uncorrected *p*-values < 0.05.

## Data Availability

The required data are available from corresponding author in reasonable request.

## Abbreviations

AD: Axial diffusivity
CSTC: Cortico-striato-thalamo-cortical
DSI: Diffusion spectrum imaging
DTI: Diffusion tensor imaging
DLPFC: Dorsolateral prefrontal cortex
FDR: False discovery rate
FS: Frontostriatal
GFA: Generalized fractional anisotropy
GTS: Gilles de la Tourette syndrome
MD: Mean diffusivity
RD: Radial diffusivity
TBAA: Tract-based automatic analysis
VLPFC: Ventrolateral prefrontal cortex
