# Supplementary material for: The microstructural change of the brain and its clinical severity association in pediatric Tourette syndrome patients
Source: J Neurodev Disord. 2023 Oct 25;15:34. doi: 10.1186/s11689-023-09501-0 (PMC10598924; doi:10.1186/s11689-023-09501-0)
Supplement: Supplementary file 1 — Additional file 1: Supplementary Table 1. The result of whole brain comparison. Supplementary Table 2. The result of comparison within CSTC circuit. The listed neural fiber tracts include only those with uncorrected p-values < 0.05. [file 11689_2023_9501_MOESM1_ESM.docx]

**Supplementary Tables**

**Supplementary Table 1**. The result of whole brain comparison

| **GFA value** |  |  |  |  |
| --- | --- | --- | --- | --- |
| **Tract Name** | **TS Group** | **HC group** | **p-value** | **q-value** |
| Left arcuate fasciculus | 0.404 + 0.034 | 0.382 + 0.033 | 0.017 | 0.215 |
| Right cingulum of main body component | 0.454 + 0.055 | 0.420 + 0.057 | 0.024 | 0.223 |
| Right frontal aslant tract | 0.355 + 0.032 | 0.338 + 0.032 | 0.042 | 0.223 |
| Right perpendicular fasciculus | 0.313 + 0.033 | 0.299 + 0.022 | 0.048 | 0.228 |
| Left FS tract of VLPFC | 0.313 + 0.032 | 0.297 + 0.023 | 0.037 | 0.223 |
| Right FS tract of VLPFC | 0.316 + 0.033 | 0.299 + 0.029 | 0.036 | 0.223 |
| Right FS tract of DLPFC | 0.452 + 0.031 | 0.430 + 0.029 | 0.007 | 0.167 |
| Right FS tract of precentral gyrus | 0.406 + 0.028 | 0.386 + 0.026 | 0.006 | 0.167 |
| Left thalamic radiation of VLPFC | 0.386 + 0.028 | 0.372 + 0.024 | 0.035 | 0.223 |
| Right thalamic radiation of VLPFC | 0.392 + 0.028 | 0.377 + 0.027 | 0.044 | 0.223 |
| Left thalamic radiation of DLPFC | 0.483 + 0.022 | 0.464 + 0.031 | 0.011 | 0.167 |
| Right thalamic radiation of DLPFC | 0.479 + 0.025 | 0.460 + 0.029 | 0.009 | 0.167 |
| Left thalamic radiation of auditory nerve | 0.265 + 0.024 | 0.253 + 0.018 | 0.032 | 0.223 |
| Corpus callosum of DLPFC | 0.478 + 0.037 | 0.451 + 0.040 | 0.01 | 0.167 |
| Corpus callosum of VLPFC | 0.463 + 0.027 | 0.445 + 0.038 | 0.036 | 0.223 |
| Corpus callosum of superior temporal gyrus | 0.493 + 0.027 | 0.477+ 0.030 | 0.043 | 0.223 |
| **RD value** |  |  |  |  |
| **Tract Name** | **TS Group** | **HC group** | **p-value** | **q-value** |
| Right cingulum of main body component | 0.346 + 0.037 | 0.368 + 0.041 | 0.042 | 0.304 |
| Right frontal aslant tract | 0.385 + 0.029 | 0.403 + 0.028 | 0.018 | 0.304 |
| Left uncinate fasciculus | 0.465 + 0.031 | 0.482 + 0.033 | 0.048 | 0.304 |
| Left FS tract of VLPFC | 0.393 + 0.026 | 0.406 + 0.023 | 0.039 | 0.304 |
| Left FS tract of DLPFC | 0.319 + 0.021 | 0.331 + 0.025 | 0.043 | 0.304 |
| Right FS tract of DLPFC | 0.307 + 0.022 | 0.324 + 0.025 | 0.009 | 0.304 |
| Left thalamic radiation of VLPFC | 0.369 + 0.023 | 0.384 + 0.023 | 0.02 | 0.304 |
| Left thalamic radiation of DLPFC | 0.303 + 0.019 | 0.317 + 0.028 | 0.024 | 0.304 |
| Right thalamic radiation of DLPFC | 0.300 + 0.020 | 0.314 + 0.024 | 0.025 | 0.304 |
| Corpus callosum of DLPFC | 0.313 + 0.026 | 0.332 + 0.030 | 0.012 | 0.304 |

DLPFC: dorsolateral prefrontal cortex, FS: frontal-striatal, GFA: generalized fractional anisotropy, RD: radial diffusivity, VLPFC: ventrolateral prefrontal cortex

**Supplementary Table 2.** The result of **c**omparison within CSTC circuit.

| **GFA value** |  |  |  |  |  |
| --- | --- | --- | --- | --- | --- |
| **Tract Name** | **TS Group** | **HC group** | **p-value** | **q-value** |  |
| Left FS tract of VLPFC | 0.313 + 0.032 | 0.297 + 0.023 | 0.037 | 0.085 |  |
| Right FS tract of VLPFC | 0.316 + 0.033 | 0.299 + 0.029 | 0.036 | 0.085 |  |
| Right FS tract of DLPFC | 0.452 + 0.031 | 0.430 + 0.029 | 0.007 | 0.044* |  |
| Right FS tract of precentral gyrus | 0.406 + 0.028 | 0.386 + 0.026 | 0.006 | 0.044* |  |
| Left thalamic radiation of VLPFC | 0.386 + 0.028 | 0.372 + 0.024 | 0.035 | 0.085 |  |
| Right thalamic radiation of VLPFC | 0.392 + 0.028 | 0.377 + 0.027 | 0.044 | 0.088 |  |
| Left thalamic radiation of DLPFC | 0.483 + 0.022 | 0.464 + 0.031 | 0.011 | 0.044* |  |
| Right thalamic radiation of DLPFC | 0.479 + 0.025 | 0.460 + 0.029 | 0.009 | 0.044* |  |
| **RD value** | |  |  |  |  |
| **Tract Name** | | **TS Group** | **HC group** | **p-value** | **q-value** |
| Left FS tract of VLPFC | | 0.393 + 0.026 | 0.406 + 0.023 | 0.039 | 0.11 |
| Left FS tract of DLPFC | | 0.319 + 0.021 | 0.331 + 0.025 | 0.043 | 0.11 |
| Right FS tract of DLPFC | | 0.307 + 0.022 | 0.324 + 0.025 | 0.009 | 0.1 |
| Left thalamic radiation of VLPFC | | 0.369 + 0.023 | 0.384 + 0.023 | 0.02 | 0.1 |
| Left thalamic radiation of DLPFC | | 0.303 + 0.019 | 0.317 + 0.028 | 0.024 | 0.1 |
| Right thalamic radiation of DLPFC | | 0.300 + 0.020 | 0.314 + 0.024 | 0.025 | 0.1 |

DLPFC: dorsolateral prefrontal cortex, FS: frontal-striatal, GFA: generalized fractional anisotropy, RD: radial diffusivity, VLPFC: ventrolateral prefrontal cortex
